# Whole genome comparative analysis of transposable elements provides new insight into mechanisms of their inactivation in fungal genomes

**DOI:** 10.1186/s12864-015-1347-1

**Published:** 2015-02-28

**Authors:** Joëlle Amselem, Marc-Henri Lebrun, Hadi Quesneville

**Affiliations:** INRA, UR1164 URGI Research Unit in Genomics-Info, F-78026 Versailles, France; INRA, UR1290 BIOGER, Biologie et gestion des risques en agriculture, Campus AgroParisTech, F-78850 Thiverval-Grignon, France

**Keywords:** Transposable elements, Fungi, Repeat induced point mutation, C5-methyltransferase

## Abstract

**Background:**

Transposable Elements (TEs) are key components that shape the organization and evolution of genomes. Fungi have developed defense mechanisms against TE invasion such as RIP (Repeat-Induced Point mutation), MIP (Methylation Induced Premeiotically) and Quelling (RNA interference). RIP inactivates repeated sequences by promoting Cytosine to Thymine mutations, whereas MIP only methylates TEs at C residues. Both mechanisms require specific cytosine DNA Methyltransferases (RID1/Masc1) of the Dnmt1 superfamily.

**Results:**

We annotated TE sequences from 10 fungal genomes with different TE content (1-70%). We then used these TE sequences to carry out a genome-wide analysis of C to T mutations biases. Genomes from either Ascomycota or Basidiomycota that were massively invaded by TEs (*Blumeria*, *Melampsora*, *Puccinia*) were characterized by a low frequency of C to T mutation bias (10-20%), whereas other genomes displayed intermediate to high frequencies (25-75%). We identified several dinucleotide signatures at these C to T mutation sites (CpA, CpT, and CpG). Phylogenomic analysis of fungal Dnmt1 MTases revealed a previously unreported association between these dinucleotide signatures and the presence/absence of sub-classes of Dnmt1.

**Conclusions:**

We identified fungal genomes containing large numbers of TEs with many C to T mutations associated with species-specific dinucleotide signatures. This bias suggests that a basic defense mechanism against TE invasion similar to RIP is widespread in fungi, although the efficiency and specificity of this mechanism differs between species. Our analysis revealed that dinucleotide signatures are associated with the presence/absence of specific Dnmt1 subfamilies. In particular, an RID1-dependent RIP mechanism was found only in Ascomycota.

**Electronic supplementary material:**

The online version of this article (doi:10.1186/s12864-015-1347-1) contains supplementary material, which is available to authorized users.

## Background

Transposable elements (TEs) are mobile genetic element able to transpose and multiply in genomes. A unified TE classification with further subdivisions into subclasses, orders and families has been proposed [1]. The two major subdivisions are class I (retrotransposons) and class II (DNA transposons), which differ according to their mechanism of transposition. Massive TE expansions play a significant role in genome structure, dynamics and evolution [2]. They shape the genomic landscape by providing novel DNA sequences at various locations, by contributing to chromosomal rearrangements, gene duplications, gene loss and inactivation and by accelerating evolution. Thus, TEs play an important role in adaptation and speciation [3]. In fungi, they have been shown to accelerate the evolution of genes that affect pathogenicity and host range [4,5]. The ability of TEs to invade genomes is frequently counterbalanced by defense mechanisms that restrain their expression and mobility [6]. Three defense mechanisms against TEs are known in fungi [7]. Repeat-Induced Point Mutation (RIP) occurs at a premeiotic stage during sexual reproduction. This defense mechanism detects DNA sequence duplications and induces irreversible C:G to T:A mutations at a high rate in these sequences. This fungal-specific defense mechanism was first discovered in *N. crassa* [8,9] and then in a few other fungal species [10]. RIP occurs preferentially at CpA dinucleotide sites in most fungi [9], although other dinucleotide sites including CpG may also be involved [11-13]. RIP requires the *RID1* gene, predicted to encode a C5-DNA-Methyltransferase (MTase) of the Dnmt1 family [14]. Dnmt1 enzymes methylate cysteine residues at specific dinucleotide sites (mainly CpG sites) [15]. The high number of C to T mutations associated with RIP is thought to occur by *RID*1-mediated deamination of methylated cytosine, leading to its replacement with thymine [10,16]. Orthologs of this gene have been identified in other *Neurospora* species [16] including *Aspergillus fumigatus* and *Aspergillus nidulans* [17], *Fusarium graminearum* [18], and *Leptosphaeria maculans* [19]. The second defense mechanism, called MIP (Methylation Induced Premeiotically), also occurs during sexual reproduction. It was first discovered in *Ascobolus immersus* and is required for the *de novo* methylation of cytosine in repeated sequences during meiosis. MIP requires *MASC1*, a gene encoding a putative a Dnmt1 cytosine methyl transferase related to *RID1* [20-22]. A third defense mechanism, called Quelling, was first identified in *N. crassa* [23]. It involves the RNA interference machinery (AGO, RDE) that suppresses TE expression [24,25].

Another gene-silencing process, the meiotic silencing by unpaired DNA (MSUD) occurring in meiotic cells has been reported. In *N. crassa*, unpaired DNA causes silencing of all their homologs DNA (paired or not). This process is mediated by putative RNA-directed RNA polymerase (RDRP) [26].

Both RIP and MIP require a Dnmt1 cytosine methyl transferase (MTase) from the same subfamily [14]; therefore, these processes probably rely on similar molecular mechanisms, such as transient *de novo* methylation of cytosine residues in repeated sequences during sexual reproduction at specific dinucleotide sites. Other known fungal Dnmt1 MTases are involved in cytosine methylation associated with heterochromatin maintenance such as Dim-2 in *N. crassa* [27] and Masc2 in *A. immersus* [28], both of which belong to a different Dnmt1 subfamily than RID1/Masc1 [22,28]. Dnmt1 MTases normally methylate at CpG dinucleotides [28]. However, Dnmt1 MTases involved in RIP bias may have evolved to recognize dinucleotides other than CpG. In the basidiomycete *Microbotryum violaceum*, C to T mutation bias in TEs occurs preferentially at a trinucleotide TpCpG site [29], suggesting the involvement of a Dnmt1 MTase specific to the CpG di-nucleotide. This mutation bias at CpG di-nucleotide is consistent with the model of mutation via methylation-mediated deamination of 5-methyl cytosine (5mC) previously describe in human genetic disease [30]. In Ascomycetes, RIP occurs preferentially at dinucleotide CpA sites [13]; however, in *A. niger* and *A. fumigatus* RIP may also occur at CpG sites in TEs in addition to CpA [11-13], suggesting the involvement of Dnmt1 Mtases recognizing mainly CpA but also CpG di-nucleotides. However it has also been suggested that the RIP-like process based on 5mC methylation followed by deamination may operate in a different manner in basidiomycetes than ascomycetes and that the *rid* homologue may have diverged during the evolutionary time separating these two phyla [31].

In this study, we conducted a bioinformatics analysis of C to T mutation bias in 10 fungal genomes with different TE content, which were recently analysed using the TEdenovo [32,33] and the TEannot [34] from the REPET TE annotation package in the context of international fungal genome projects. We used the consensus sequences of TE families and corresponding TE genomic sequences for each genome obtained from the TEdenovo and the TEannot pipelines, respectively. We performed (i) a genome-wide comparison of TE content; (ii) a RIPCAL-based [35] exhaustive search of C to T mutations in TEs with their associated dinucleotide sites; and (iii) a functional annotation and phylogenetic analysis of genes encoding RID/Masc1 and Dim-2/Masc2 Dnmt1 MTases in these 10 genomes. The observed dinucleotide patterns at C to T mutation sites in fungal TEs suggest that TE defense mechanisms have different sequence specificities. Our findings also reveal an association between these patterns and the presence of specific Dnmt1 genes. We propose a scenario for the evolution of Dnmt1 subfamilies, in which the RID1-dependent RIP mechanism emerged in Ascomycota after its radiation from Basidiomycota.

## Results

### Recent TE invasions in fungi explain the lack of correlation between genome size and taxonomy

**Figure 1:**
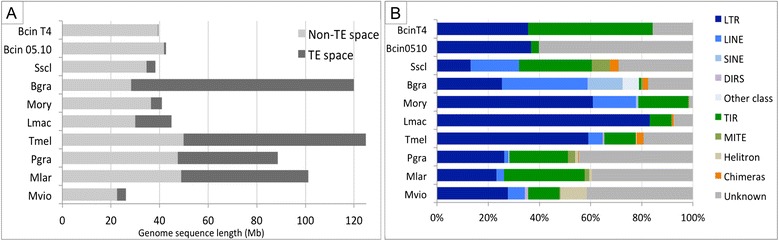
**Distribution of TEs in fungal genomes. (A)** TE vs non-TE space, **(B)** Percentage of different categories of TEs: class I retrotransposons (LTR, LINE, SINE, DIRS) and class II DNA transposons (TIR, MITE, Helitrons), unclassified (Unknown) and chimeras of TE consensus sequences.

We used the REPET TE annotation package to produce a standardized genome-wide annotation of TEs from 10 fungi (Table 1, Additional file 1) with different lifestyles, including seven ascomycetes (*Botrytis cinerea* (T4 and B05.10 isolates), *Sclerotinia sclerotiorum* [36], *Blumeria graminis* fp. hordeï [37], *Leptosphaeria maculans* [19], *Magnaporthe oryzae* [38], *Tuber melanosporum* [39]) and three basidiomycetes (*Puccinia graminis* and, *Melampsora larici-populina* [40] and *Microbotryum violaceum* (in prep; *Microbotryum violaceum* Sequencing Project, http://www.broadinstitute.org). Among the 10 species studied, BcinT4 (genome abbreviation see Table 1) had the lowest TE content (1% of its genome), whereas Bgra had the highest (75% of its genome Table 1, Figure 1A). Sscl, Mory and Mvio had intermediate TE content (9 to 15%), close to the average TE content in fungi (25%, [40,41]). Lmac, Tmel, Pgra, Mlar had high TE content (35-50%). The genomes of fungi with high TE content (>35%) were larger than those of typical fungi or related species with low TE content (Figure 1A). The large TE content of Bgra (75%) substantially affects the size and organization of its genome: the Bgra genome is 120 Mb, which is 80 Mb larger than that of the related species Bcin and Sscl, contains only 30 Mb of coding space, compared to 40 Mb in other Ascomycetes species, and many of its genes are surrounded by TE rich regions. The highly related species *Blumeria graminis* formae speciales *tritici* also has a genome with high TE content (90%) as do other related species from the Erysiphales (80-90%) [42]. The occurrence of TE functional categories greatly differs between species (Figure 1B) and is independent of their taxonomic relationship (Table 1), except for the related rust fungi Pgra and Mlar [40]. For example, Tmel, Mory and Lmac are characterized by a high proportion of LTR retrotransposons (60-80% of TE content), although they are classified in very distantly related taxonomic orders, whereas the main TE categories in more related species such as Mory and Sscl are very different: LTRs make up 10% and 60% of TEs in Sscl and Mory, respectively and TIR TEs make up 30% and 20% of TEs in Sscl and Mory, respectively (Figure 1B). We noted similar differences between highly related species such as Bcin and Sscl. LINE and Helitron TEs were detected in Sscl (15% and 5% of TE content, respectively), but not in Bcin, whereas the proportion of LTR TEs was lower in Sscl (10%) than in Bcin (36%). The differences in TE content and TE categories among related species, such as BcinT4 and Bcin0510, and Sscl and Bgra, are presumably due to relatively recent TE invasions, as suggested previously [36,37,39]. For example, differences in the composition of TEs between Sscl and Bcin are due to the recent invasion of a few families of class II TIR DNA and LINE TEs in Sscl. These subfamilies of Sscl TIR TEs are composed of almost identical copies dispersed in the genome, whereas other subfamilies of Sscl TIR TEs are highly polymorphic [36].

**Table 1:** **Taxonomy of fungal genomes, source and lifestyle**

**Fungi kingdom**	**Strain**	**Abrev.**	**Phylum**	**Class Family**	**NCBI BioProject**	**% TE**	**Assembly size**	**Lifestyle**
*Botrytis cinerea* T4	T4	BcinT4	Ascomycota	Leotiomycetes Sclerotiniaceae	64593	0.7	39.5 Mb	Necrotrophic pathogen
*Botrytis cinerea* 05.10	05.10	Bcin0510		Leotiomycetes Sclerotiniaceae	20061	2.2	38.8 Mb	Necrotrophic pathogen
*Sclerotinia sclerotiorum*	1980 UF-70	Sscl		Leotiomycetes Sclerotiniaceae	20263	9.5	38.2 Mb	Necrotrophic pathogen
*Blumeria graminis f. sp. hordeï*	DH14	Bgra		Leotiomycetes Erysiphaceae	28821	76.4	120 Mb	Obligate biotrophic pathogen
*Magnaporthe oryzae*	7015	Mory		Sordariomycetes Magnaporthaceae	1433	11.1	40.9 Mb	Hemibiotrophic pathogen
*Leptosphaeria maculans*	JN3	Lmac		Dothideomycetes	63129	33.3	44.9 Mb	Hemibiotrophic pathogen
*Tuber melanosporum*	Mel28	Tmel		Pezizomycetes Leptosphaeriaceae	49017	60.1	123.6 Mb	Symbiotic
*Melampsora larici-populina*	98AG31	Mlar	Basidiomycota	Pucciniomycetes Melampsoraceae	46711	51.7	101.1 Mb	Obligate biotrophic pathogen
*Puccinia graminis f. sp. tritici*	CRL 75-36-700-3	Pgra		Pucciniomycetes Pucciniaceae	66375	46.4	81.6 Mb	Obligate biotrophic pathogen
*Microbotryum violaceum*	P1A1 Lamole	Mvio		Pucciniomycetes Mycrobotryaceae	41281	14.1	25.2 Mb	Obligate biotrophic pathogen

### Analysis of TE G:C content reveals multi-modal distributions

**Figure 2:**
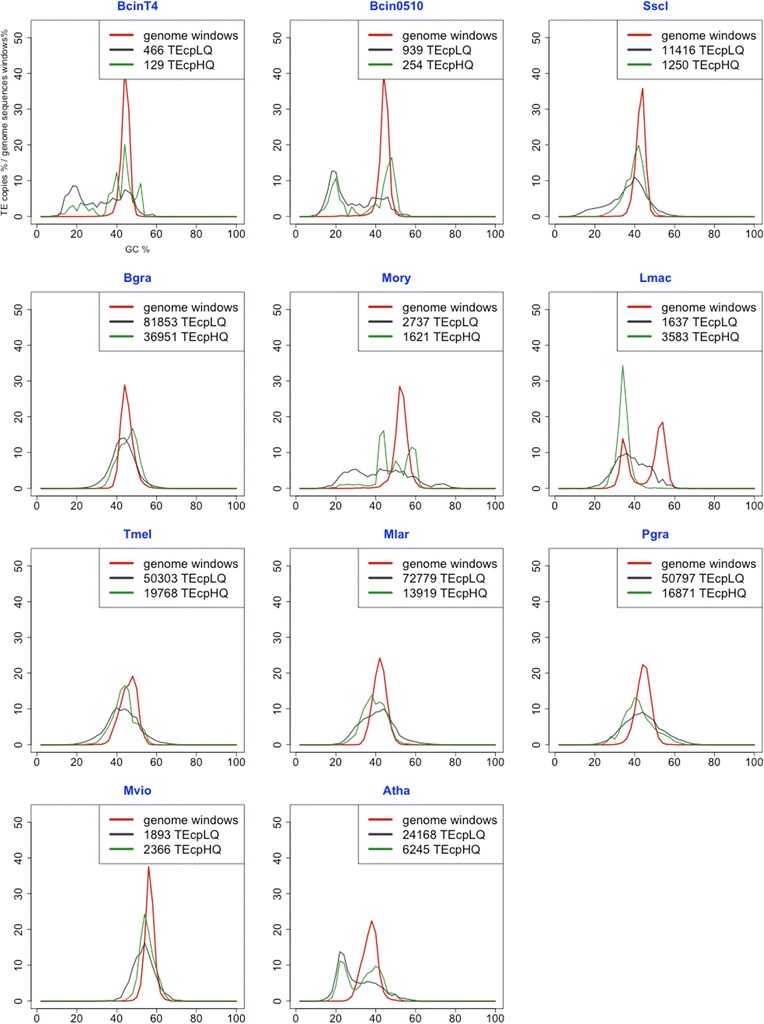
**Comparison of GC content (%) in TE copies and genome sliding windows.** GC content in TE copies: GC content in Low quality (TEcpLQ, gray) and High quality (TEcpHQ, green) TEs and in genome sliding windows (red) was calculated with sliding windows (windows = 2 Kb, increment = 2 Kb). Y axis: TE copies% or Sliding windows%. X axis: GC%.

One major consequence of RIP involves genome G:C content, because RIP increases the A:T content of mutated TE copies. As a result, when TEs are clustered in large blocks, the RIP-mediated mutation of C:G to A:T generates A:T rich isochores, as observed in Lmac [19]. We sought to identify A:T rich regions associated with TEs; therefore, we compared the G:C content of high quality TEs (see Methods, hereafter referred to as TEcpHQ) or low quality TEs (hereafter referred to as TEcpLQ) with the G:C content of the whole genome across 2 Kb sliding windows denoted as GSW. Lmac was the only species of our sample that displayed a bimodal GSW. The peak at 36% G:C corresponds to A:T-rich isochores composed mainly of RIPed TE copies (Figure 2), which have been described previously [19]. Other genomes displayed a unimodal GSW with a peak around 45-50% G:C, with the exception of Mvio (60%) and the negative control Atha (42%). The distribution of G:C content in TEs frequently differed from that in the GSW. In the negative control Atha, the G:C content of TEcpHQ was bimodal. The low TEcpHQ values (20%) probably correspond to heavily mutated ancestral TEs that tend to be depleted in C:G sites because they are highly methylated [43,44]. In genomes invaded by TEs (Mvio, Tmel, Mlar, Pgra, Bgra) the distribution of G:C content was similar between TEs (TEcpHQ) and the whole genome (GSW, Figure 2). However, the high TE content of these genomes clearly introduces a bias because TE content makes up a larger proportion of genomic space than non-TE content. In other genomes, the distribution of G:C content between TEs and the whole genome was very different, in particular for TEcpHQ. For example, TEcpHQ elements in BcinT4 had four peaks of GC content; one of these peaks (44% G:C) was very similar to the whole genome (GSW) peak, whereas the three other peaks comprised two groups of TEs with low G:C content (20 and 40%) and one group with high G:C content (55%). Mory showed a similar profile consisting of two peaks (40 and 60%) surrounding another peak at 50% corresponding to the whole genome GC content. TEs peaks with low G:C content in BcinT4 may correspond to TEs copies that have undergone RIP-associated C to T mutations at various rates. Indeed, the TEcpHQ peak at 20% G:C content in BcinT4 comprises AT-rich TE copies that are even more abundant among TEcpLQ elements (Figure 2). These highly degenerated TEs may result from multiple rounds of RIP leading to the mutation of all their target C:G sites.

### Dinucleotide signatures associated with C to T mutation sites in TEs differ among fungi and TE families

**Figure 3:**
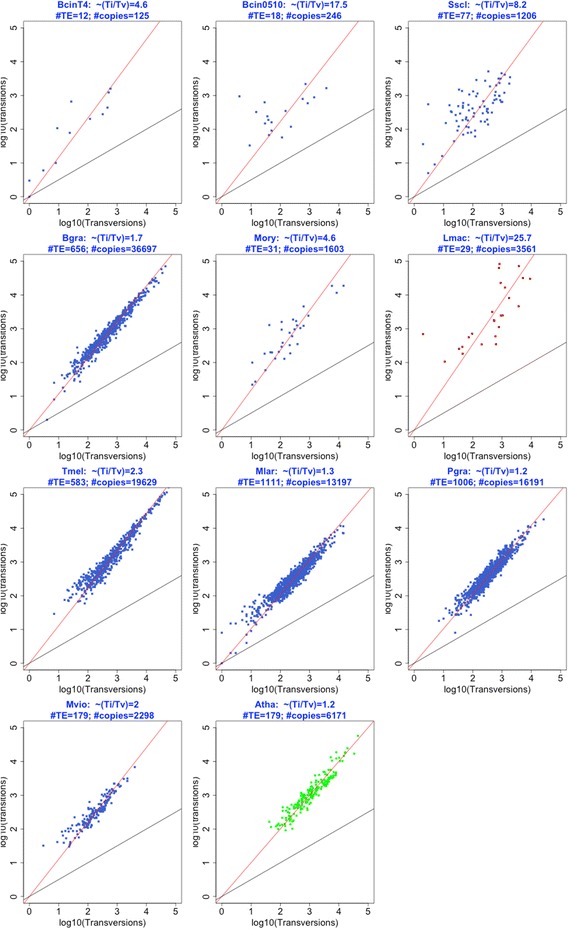
**Transition and transversion mutation rates in TE copies from fungal genomes.** Transition (Ti, Y axis, log10) and Transversion (Tv, X axis, log10) . Mutation rates were calculated using RIPCAL by comparing each TE copy with the TE consensus sequence or the genomic copy with the highest GC content through multiple alignments. Each dot corresponds to the log10(sum Ti) vs. log10(sum Tv) of a TE family. ~(Ti/Tv) is the mean of Ti/Tv for all the families in the analysis. The black line corresponds to the linear regression line between the two series (Ti vs Tv). The red line corresponds to an equiprobable mutation rate (Ti/Tv = 0.5). The Lmac and Atha dot plots are red and green respectively to highlight the pattern of positive and negative controls respectively.

**Figure 4:**
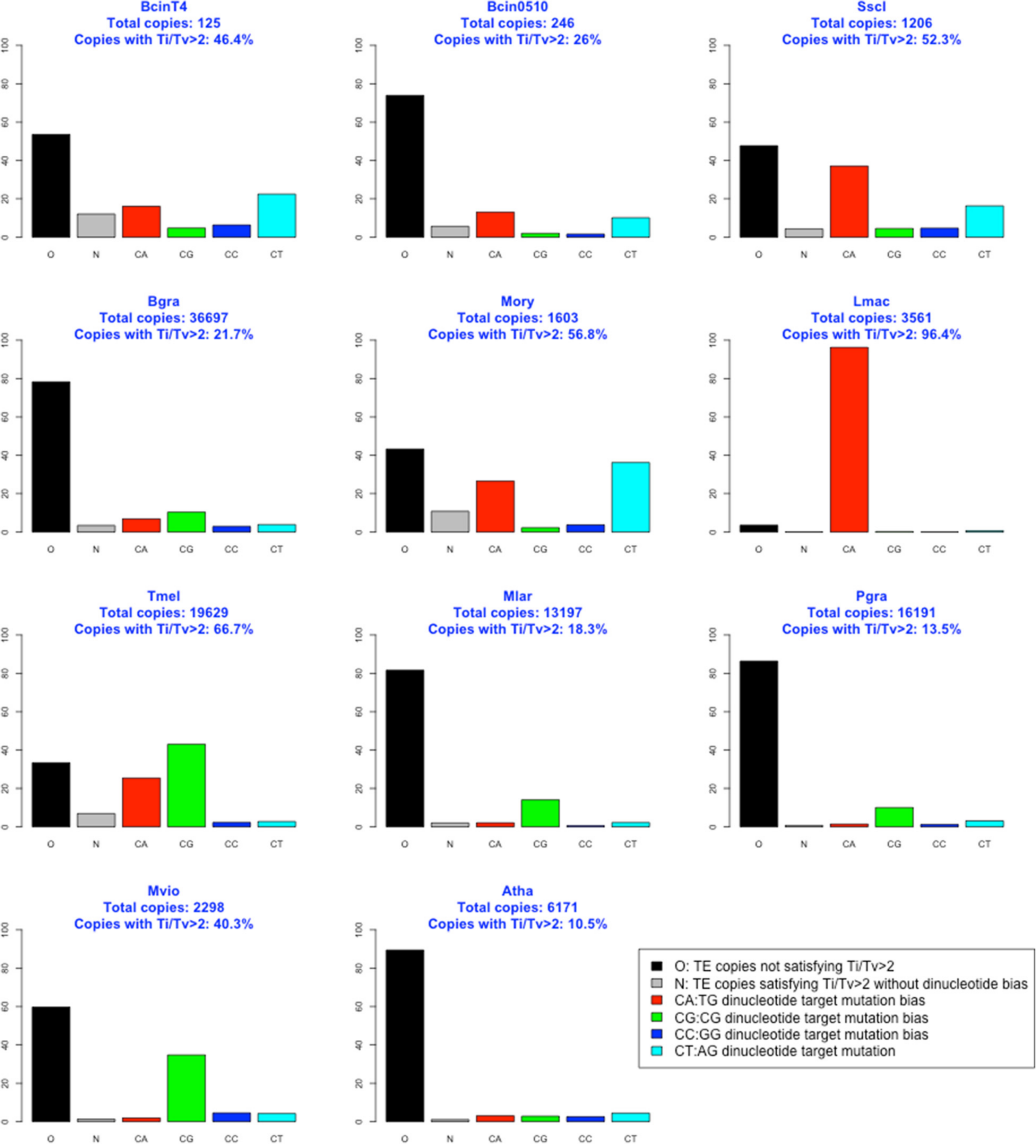
**Distribution of TE copies according to their dinucleotide mutation bias.** Mutation rates were calculated using RIPCAL by comparing each TE copy with a Ti/Tv > 2 with the TE consensus sequence or the full-length genomic copy with the highest GC content through multiple-alignments. Y-axis: percentage relative to the total number of copies used in RIPCAL analysis. - Colored bars correspond to the percentage of copies with expected RIP* and dinucleotide preferentially used (>1/3) in CN- > TN and (cNG - > cNA) mutations. - Black bar: percentage of copies without expected RIP* (Ti/Tv > 2). - Gray bar: percentage of copies with expected RIP* but no evidence of dinucleotide bias. * RIP expected: Ti/Tv > 2. Note: more than one dinucleotide bias could be observed in a single TE copy.

The mutation resulting from RIP is a transition (C:G to T:A). We used multiple sequence alignment (MSA) to compare genomic TE copies with the consensus TE sequence of each family to search for mutational biases and identify RIP-associated signatures in fungal TEs. If all possible mutations were equally likely, we would expect twice as many transversions (Tv) as transitions (Ti), given a random mutation Ti/Tv ratio of 0.5. We used MSAs from Atha TEs [32,45] as negative controls of RIP-associated biases because RIP has not been observed in plants. We found that the mean Ti/Tv ratio of Atha TE families was equal to 1.2 (all TEs, Figure 3). Genome-wide analysis of Bgra, Pgra and Mlar TEs showed that their average Ti/Tv ratio was similar to that of Atha (all TEs = 1.2 to 1.7). TEs copies from all other fungal genomes displayed a higher mean Ti/Tv ratio (all TEs = 2 to 25.7). We selected TE genomic copies with a Ti/Tv > 2 (Figure 3), which is a stringent threshold compared to the Atha negative control (Ti/Tv =1.2). Using these TE sequences, we searched for dinucleotides associated with C to T mutation sites. Lmac TEs were used as a positive control for a RIP-associated signature [19]. We first observed that the percentage of TE copies with a high Ti/Tv ratio varied widely from genome to genome (Figure 3). Most Lmac TEs (96%) had a high Ti/Tv ratio (Ti/Tv > 2, Figure 3), as reported previously [19]. By contrast, the percentage of TEs with a Ti/Tv ratio > 2, was low (14 to 22%) and similar to that of the negative control Atha (11%) in the three species (Bgra, Mlar, Pgra) massively invaded by TEs, suggesting that they are deficient for RIP. The remaining species had an intermediate proportion of TEs (40 to 67%) with a Ti/Tv ratio > 2 (Figure 3). We next searched for dinucleotide signatures at C to T mutation sites in TEs with a Ti/Tv ratio > 2 (Figure 4). As expected, we found that the vast majority (90%) of Lmac TE C to T mutation sites was associated with a CpA dinucleotide (Figure 4). We also found this canonical RIP-associated dinucleotide signature in a large fraction (40%) of Sscl TEs. However, we identified other dinucleotide signatures (CpT, CpG) at C to T mutation sites in TEs from other fungal genomes. In some fungal species, these were the only signatures present in TEs, whereas in other species they were found in addition to CpA signatures. In BcinT4, BcinB0510, Sscl, and Mory, we identified a large number of TEs with a CpT signature (10-40% of TEs) in addition to CpA signatures (10-35% of TEs). In the TE-rich Tmel genome, many TE copies (67%) displayed a Ti/Tv ratio > 2. The C to T mutation sites of Tmel TEs displayed predominantly a CpG signature (40% of TEs) in addition to a CpA signature (25% of TEs). In Mvio, a large number of TE copies (40%) displayed a Ti/Tv ratio > 2. The C to T mutation sites of Mvio TEs displayed only a CpG signature (37% of TEs, Figure 3). In genomes with high TE content (Bgra, Pgra and Mlar), the number of TE copies that displayed only a CpG signature at their C to T mutation site was small (Figure 4).

### Dinucleotide signatures at C to T mutation sites in fungal TEs are associated with specific Dnmt1 subfamilies

**Figure 5:**
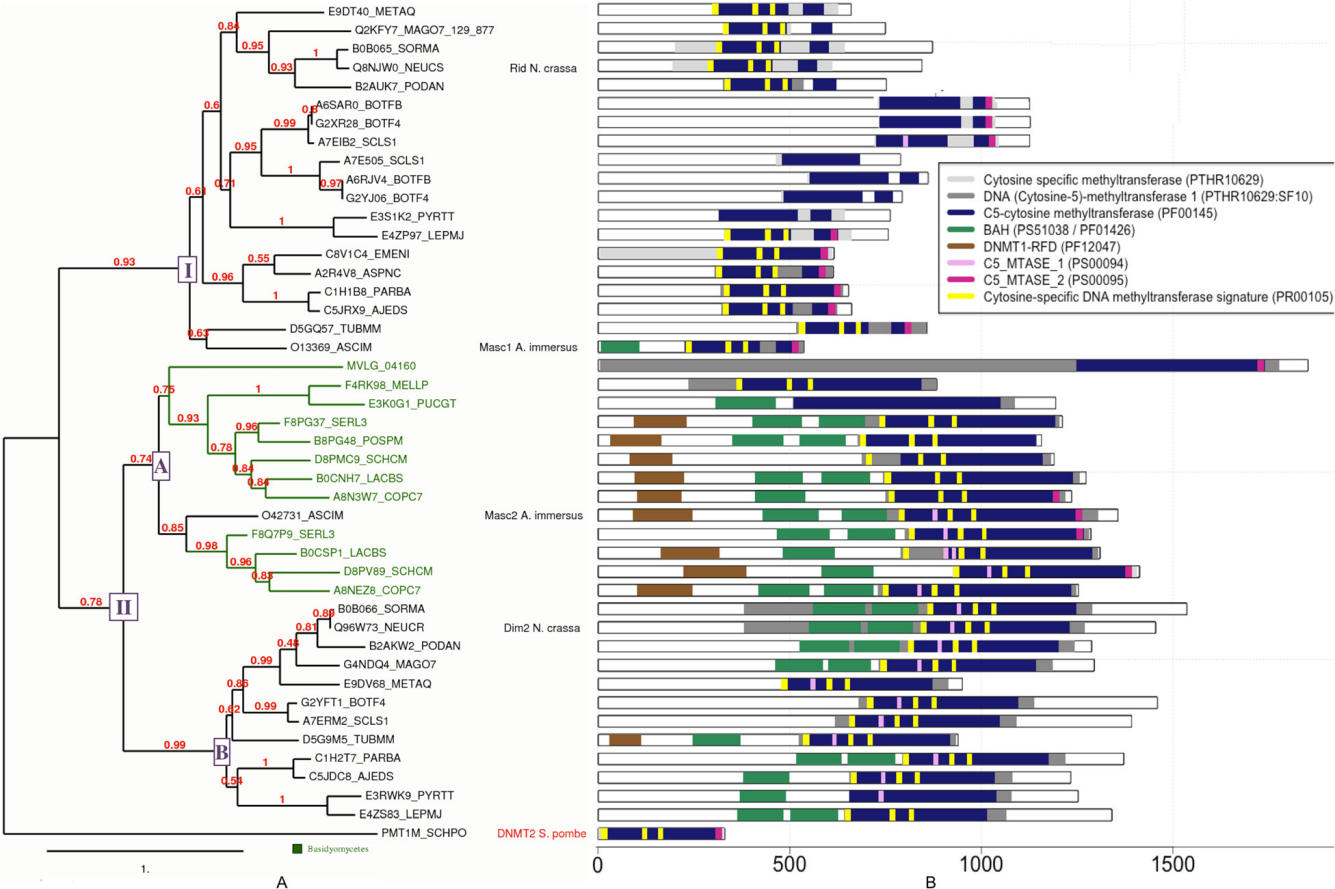
**Functional annotation of 44 fungal Dnmt1 proteins and phylogenetic analysis of the cytosine-specific methyltransferase domain. (A)** Phylogenetic analysis of 44 cytosine-specific methyltransferase domains (PTHR10629): Gray rectangles in 5B, including PF00145, PS00094, PS00095 et PR00105) from Dnmt1 fungal proteins and *S. Pombe* DNMT2, which was used as an outgroup. **(B)** Functional annotation of genes (white rectangle) with Interproscan (Cf Methods section). PTHR10629:SF10 is drawn on top of PTHR10629 (the coordinates are the same). Where PTHR10629 is not visible, it was overlapped by PF00145.

We next addressed whether the differences in C to T mutation bias in TEs and their associated dinucleotide signatures among fungi could be explained by the presence/absence in these various species of different genes involved in this process. RIP and MIP require cytosine methyltransferases of the Dnmt1 family; therefore, we searched for 25 fungal genomes (included the 10 fungal genomes studied) for genes encoding Dnmt1 MTases. Forty-four proteins containing the cytosine specific methyltransferase Panther domain (PTHR10629) were recovered from these 25 genomes. This domain overlaps on both ends the C5-cytosine methyltransferase PFAM domain (PF00045), which is specific to the cytosine DNA MTase family (Figure 5B). Phylogenetic analysis of the PTHR10629 domain extracted from the 44 Dnmt1 protein sequences (Figure 5A) showed that these proteins clustered into two very distinct clades. The first clade (class I), contains proteins related to RID and Masc1 whereas the second clade (class II) contains proteins related to Dim2 and Masc2. This second clade is composed of two subclasses (IIA and IIB), corresponding to proteins related to either Masc2 or to Dim2, respectively. Interestingly, all the genomes with CpA or CpT dinucleotide signatures in TEs, had at least one gene coding for a protein of the Masc1/RID family (Dnmt1 class I, Figure 5A, Table 2). All these genomes belong to species of Ascomycota (Additional file 1). The three genomes that displayed only TEs with a CpG dinucleotide signature (Mvio, Mlar and Pgra) contained only one gene coding for a Dnmt1, a class IIA protein related to Masc2 (Figure 5A, Table 2). These three organisms belong to the Basidiomycota.

**Table 2:** **Association between dinucleotide signatures preferentially found at C:G to T:A mutation sites and presence/absence of DNMT1 genes**

**Organism**	**Predominant**	**Secondary**	**Dnmt1 class I**	**Dnmt1 class IIA**	**Dnmt1 class IIB**
	**C to T**	**C to T**	**RID/Masc1**	**Masc2**	**Dim2**
	**Dinucleotide mutation bias**	**Dinucleotide mutation bias**	**Uniprot accession no. of similar protein**	**Uniprot accession no. of similar protein**	**Uniprot accession no. of similar protein**
Lmac	CpA	-	E4ZP97_LEPMJ	Nd	E4ZS83_LEPJM
Mory	CpT	CpA	Q2KFY7_MAG07	Nd	Nd
BcinT4	CpT	CpA	G2XR28_BOTF4	Nd	G2YFT1
			G2YJ06_BOTF4		
BcinB05.10	CpA	CpT	A6SAR0_BOTBB	Nd	Nd
			A6RJV4_BOTFB		
Sscl	CpA	CpT	A7E1B2_SSCL1	Nd	A7ERM2_SSCL1
			A7E505_SSCL1		
Bgra	^1^CpG	-	Nd	Nd	Nd
Tmel	CpG	CpA	D5GQ57_TUBMM	Nd	D5G9M5_TUBMM
Pgra	^1^CpG	-	Nd	E3K0G1_PUCGT	Nd
Mlar	^1^CpG	-	Nd	F4RK98_MELLP	Nd
Mvio	CpG	-	Nd	MVLG_04160	Nd

Results are shown if the proportion of copies exhibiting a mutation bias was over 10%.

^1^Very weak bias (10% of copies). Nd: not determined.

Protein domain annotation of the 44 Dnmt1 proteins revealed that the proteins belonging to the classes and subclasses established above (phylogeny based on the PTHR10629 domain) showed extensive synapomorphy in their domain composition (Figure 5B). Phylogenetic analysis and taxonomic distribution suggest that the C5_MTASE_2 C-terminal amino-acid signature (PS00095) was most likely present in the common ancestor of both classes (most parsimonious hypothesis). By contrast, the Bromo-Adjacent Homology (BAH) domain, which is commonly found in chromatin-associated proteins and usually present in a duplicated form [46], and the N-terminal Dnmt1-RFD (PF12047), which is a methyltransferase replication foci domain of the Dnmt1 protein required for methylation of the correct residue (CpG methylation site) [47], were detected only in class 2 Dnmt1 proteins (Dim2/Masc2). Several domains were irregularly distributed among fungal Dnmt1 proteins. For instance, the PROSITE domain C5_MTASE_1 (PS00094), which is part of the PF00145 domain (i.e. the PFAM equivalent of PTHR10629), was detected in almost all (11 out of 12) Dnmt1 class IIB (Dim-2) proteins, but only in a sub-cluster (comprising 4 out of 12) of class IIA proteins that are highly related to Masc2. This domain is absent in almost all (18 out of 19) Dnmt1 class I (Masc1/RID proteins). By contrast, C5_MTASE_2 (PS00095), a C-5 cytosine-specific DNA methylase C-terminal amino-acid signature, was found in 50% of the Masc1/RID proteins and in class II proteins, this domain was restricted to the Masc2-like class IIA subcluster (Figure 5). Thorough examination of the organization of the predicted DNA MTase domain (PF00145) in fungal Dnmt1 homologs revealed that it is on average smaller (240 aa) in class I proteins than in class II proteins (400 aa). This difference is due to a large region upstream from the C-terminal catalytic domain that is present only in the Dnmt1 class II proteins. Another amino-acid probably lost the gene, the C5METTRFRASE (PR00105) from the PRINTS fingerprint protein database, was present in almost all (80%) Dnmt1 proteins. Nevertheless, it is probable that these three patterns (C5_MTASE_1, C5_MTASE_2 and C5METTRFRASE) were probably present in a common ancestor of the two classes because we found relics of these motifs in all 44 proteins analyzed (Additional file 2). We also used the MEME tool to identify conserved motifs in fungal Dnmt1 proteins. This analysis revealed that 19 out of 30 conserved motifs where specific to either Dnmt1 class I or class II proteins: five motifs (15, 17, 20, 26, 29) were present only in Dnmt1 class I proteins whereas the other 14 motifs were present only in Dnmt1 class II proteins (Additional file 3). The five motifs specific to Dnmt1 class I proteins are not part of any known functional domain. Among these five motifs specific to RID/Masc1-like proteins, one (motif 29) was only found in Bcin, Sscl and Mory (Additional file 3). This domain may be involved in the CpT di-nucleotide signature specific to these three genomes. Among the 14 motifs only present in Dnmt1 class II proteins, three (motifs 16, 27 and 28) are specific to class IIA (Masc2 proteins). Motif 16 is located in the BAH domain whereas motifs 12 and 28 are located in the C5-MTase domain. Seven other motifs are specific to the class IIB (including Dim-2) proteins (motifs 25, 22, 18, 28, 21, 30 and 19; from 5’ to 3’ respectively). Motif 21 is located in the BAH domain and motif 19 is located in the C5_MTase domain. Motifs 25, 22 and 18, which are upstream from the C5-MTase domains, are located around the motif 11 that is specific to the Dnmt1-RFD domain. This domain is present in most of class IIA proteins but only in one class IIB protein (D5G9M5_TUBMM) (Additional file 4). These results show that the evolution of sequences located in or around the DNMT1-RFD and BAH domains are compatible with the phylogenetic analysis of the C5-MTase domain.

## Discussion

### Recent TE invasions of fungal genomes explain the lack of correlation between genome size and taxonomic relationships

We were able to annotate successfully TE copies, even when they were small and degenerated. The TE consensus sequence provided by the TEdenovo detection and classification pipeline was very similar to the ancestral sequence of the TE family. The annotations obtained with these consensus libraries provided a quasi-exhaustive set of copies that could be used to search for methylation signatures of repeat-based silencing processes. Except for Pgra and Mlar, which are characterized by similar categories of TEs and an almost equivalent TE content and distribution of class I and class II elements, we did not find any correlation between the distribution of TEs and taxonomic relationships. The main difference in the composition of TEs in the two related fungi *Botrytis* and *Sclerotinia* is due to the lack of LINE elements in Bcin (Figure 1B). The difference in their TE content (8 fold higher in Sscl, Figure 1) is almost certainly due to a recent invasion of class II TIR elements [36].

### The frequency of C to T mutation bias in TEs is correlated with genome size and organization

Our RIP signature analysis in Lmac revealed the presence of AT-rich genome blocks composed of highly RIPed copies, thus confirming our previous findings in this species. Similar AT-Rich isochore-like islands were also recently found in repeated regions of the *Colletotrichum graminicola* genome [48]. These organisms may be examples of successful TE invasions (35 and 22% TE content for Lmac and *C. graminicola*, respectively) counter-balanced by an efficient RIP silencing mechanism. We also noticed that genomes with a lower TE content, such as *B. Cinerea* T4 and B0510 isolates (0.7 and 2% TE content, respectively) have many AT rich relics of TE copies that are probably signatures of ancient RIP (Figure 2, TEcpLQ). We also found an interesting correlation between fungal lifestyles and massive TE expansion; the obligate biotrophs Bgra, Mlar and Pgra and the symbiote (ectomycorrhiza) Tmel had higher TE loads than the remaining studied fungi which are all necrotrophs.

### Dinucleotide mutation signatures at C to T mutation sites in fungal TEs are associated with specific DNA methyltransferases

In four fungal species (*B. cinerea, S. sclerotiorum, M. oryzae* and *T. melanosporum*), TE copies showed mutational biases at two different dinucleotides that could be signatures of two different mechanisms (Table 2). All four species had a CpA dinucleotide signature, like in N. crassa where the RIP was experimentally observed [8] or in L. maculans where it the high level of RIP was detected in-silico [19]. *T. melanosporum* had a CpG signature, whereas a CpT signature was found in the three other species. A fifth species, *L. maculans* exhibited an extensive CpA signature suggestive of a high rate of RIP, and showed no other dinucleotide signature. A *RID/Masc1*-like gene was present (in one or two copies) only in the five species exhibiting a CpA signature in their TE copies, (which was accompanied by CpT bias in some species). We did not find any gene of the Dnmt1 family in *B. graminis f. sp. hordei*. The absence of genes responsible for repeat silencing probably explains the large expansion of TEs in this fungus, as well as in the other *formae speciale* of wheat, *B. graminis triticeae* [42]. *B. graminis* lost half of its genes during its evolution towards an obligate biotrophic lifestyle [37]. It probably also lost the gene(s) responsible for RIP. Indeed, we found traces of RIP in a very small number of TE copies that exhibit the CpA dinucleotide target signature. The RIP of these TE copies probably predates the loss of the RIP gene. Three species (*M. violaceum, M. larici-populina* and *P. graminis*) showed only a CpG mutation signature in their TE copies. In these genomes, we did not find any gene belonging to the Dnmt1 class I RID/Masc1-like phylogenetic group (Table 2); instead, we found one gene belonging the Dnmt1 class IIA (including *Masc2* gene). This suggests that Dnmt1 class IIA proteins (including Masc2) are involved in a mechanism occurring preferentially at CpG target sites. Indeed, the three species *M. violaceum, M. larici-populina* and *P. graminis* (all basidiomycetes) of our study exhibited a CpG signature (40% of Mvio TE copies and 10% of both Pgra and Mlar TE copies) and possessed a Masc2-like gene, but not any other Dnmt1-like gene. To date, no published analysis has associated the dinucleotide targets of transition mutations to potential genes and functional domains involved in these mutations.

### Evolution of Dnmt1 subgroup proteins

Here we showed that a part of the PFAM Domain PF00145 specific to DNA methyltransferase (C5_MTASE) is missing from all the RID/Masc1-like proteins (Figure 5B; Additional file 2). In addition, we found four motifs specific to these genes (Additional file 3, Motif 15, 17, 20, 26). The loss of part of the C5_MTASE domain associated with the acquisition of these four motifs may have resulted in the specialization of the gene responsible for the RIP process, the efficiency of which depends on the organism (Figure 3). A fifth motif (Additional file 3, Motif 29) only present in *B. cinerea, S. sclerotiorum* and *M. oryzae* could be involved in the recognition of the dinucleotide target CpT because this signature was detected only in TE copies from these species. The specialization of RID and Dim-2 probably occurred after the radiation between the *basidiomycota* and *ascomycota* phyla. Indeed, Masc2-like genes (class IIA phylogenic subgroup, Figure 5A) were the only Dnmt1 genes in basidiomycetes and we found neither *RID/Masc1* nor *Dim-2* genes in this phylum. This suggests that RIP does not target CpG dinucleotides. In Tmel where was found a CpG signature in addition to CpT it was recently showed that a high fraction of transposons with methylated cytosine had a strong preference for CpG sites [49]. In 2011, Clutterbuck [13] proposed that the mechanism responsible for C to T mutations at CpG target sites in fungi was probably cytosine methylation followed by the deamination of the methyl-cytosines. However a correlation between this process and the genes that are potentially involved has not yet been reported. Although we did not find any *RID*-like gene or *Dnmt1* gene in *B. graminis*, we found evidence of dinucleotide target signatures such as CpA and CpG (<10% of TE copies), showing that these genes were probably active before they were lost. Surprisingly, *Masc2* is the only gene found in an ascomycete among the class IIA group of Dnmt1 proteins. We thus hypothesize that the specialization of this gene in only one ascomycete (out of the 16) and basidiomycetes (in our phylogenetic analysis) reflects a convergent evolution (i.e. a natural selection that favors similar function in spite of different ancestor).

## Conclusions

In this article, we provide new insights into RIP and other related process involving the action of C5-Cytosine DNA methyl-transferases during repeat silencing mechanisms in fungi. The absence of RIP or its low efficiency in fungal genomes appears to be responsible for the accumulation of TEs, which increase the size of the genome. We also propose a new classification system for fungal Dnmt1 proteins based on rigorous functional annotation of domains and motifs and phylogeny of the cytosine-specific methyltransferase domain. We show that CpA and CpT dinucleotides are probably targeted by a RIP process involving the *RID* gene, whereas CpG dinucleotides are probably targeted by another methylation-based process involving a *Masc2*-like gene. The signatures of CpG to TpG mutations found in some TE copies strongly suggest a mechanism of methyl-cytosine deamination following *de novo* methylation induced by a process similar to MIP. In addition, we found that the RID protein of three species (*B. cinerea, S. sclerotiorum* and *M. oryzae*) containing signatures of CpT bias possessed a specific motif, suggesting that the *RID* gene in these three species underwent specialization to recognize this target. We propose that the RIP process and the specialization of the *RID* gene appeared after the radiation of Basidiomycota and Ascomycota phyla.

## Methods

### TE consensus and genomic copy resources

Datasets of TE families were obtained through several genome sequencing projects that we were involved in. The REPET TEdenovo pipeline [32] was used to detect TEs in genomic sequences and to provide a consensus sequence for each family. TEs were then classified according to structural and functional features (LTR, TIR, RT, transposases, polyA tail) and similarities with characterized TEs from the Repbase Update database [50]. The REPET TEannot pipeline [34] and the previously obtained TE consensus libraries were used to annotate TE genomic copies, including nested and degenerated ones, in each of the 10 genomes. Basic TE annotations have already been published for most of these species, which included: (i) six ascomycetes: *Botrytis cinerea* T4 (BcinT4 and B05.10 isolates (Bc0510) and *Sclerotinia sclerotiorum* (Sscl) [36], *Blumeria graminis* sp*. hordeï* (Bgra) [37], *Leptosphaeria maculans* (Lmac) [19] and *Tuber melanosporum* (Tmel) [39]; and (ii) three basidiomycetes: *Puccinia graminis* sp. *Triticeae* (Pgra), *Melampsora larici-populina* (Mlar) [40] and *Microbotryum violaceum* (Mvio), (in prep.). We also used TEs from *Arabidopsis thaliana* (TAIR9) that were annotated with REPET [32]. *A. thaliana* was used as negative controls of RIP-associated biases because RIP has not been observed in plants.

### TE annotation refining

Datasets of TE families (consensus + genomic TE copies) used in the reported analyses were refined in a second TEannot pipeline iteration that was carried out with the improved REPET V2 release. We filtered out poorly defined and redundant TE consensus sequences showing no full-length copies (i.e. copies covering more than 95% of the consensus sequence) in the genome. Hence, we decreased the complexity of our consensus libraries, and increased the number of TE copies found by some consensus sequences, without any significant loss of sensitivity. We also re-launched PASTEC [33] the new TE classifier implemented in the REPET V2 TEdenovo pipeline. TE consensus libraries and annotations in genomes are available at: https://urgi.versailles.inra.fr/download/fungi/TEs/.

### Search for RIP-like signatures and dinucleotide targets involved in transition mutations in genomic TE copies

We identified RIP signatures by comparing each TE genomic copy with the consensus used to annotate it. When TEs have highly diverged during evolution through a RIP-mediated irreversible process the consensus could be more “Ripped” than some of TE copies in the genome because mutations occurring in a given sequence are likely to be removed by the “base-pair majority rule” used to build the consensus. In such a case the copy with the Highest GC content is used to calculate the transition mutations. All TE families (TE consensus with their annotated TE copies) satisfying strict quality criteria were aligned (Cf section below) and processed by RIPCAL [35]. RIPCAL output provides the number of transitions (Ti), transversions (Tv) and dinucleotide targets used in all possible transitions for each TE copy. These outputs were parsed to search for RIP signatures in TE copies and the dinucleotide targets used in the transition type mutations that are usually associated with a RIP-like mechanism. In addition, R was used to generate graphics from REPET pipelines and RIPCAL results.

### Multiple sequence alignments of TE copies

Multiple sequence alignments may be difficult to calculate if TE copies are fragmented and degenerated. To tackle this problem, we first performed pairwise alignments between each copy and its cognate consensus sequence, and then we used the consensus as a reference to derive a multiple alignment using refalign and refalign2fasta respectively (from REPET package). We filtered out the consensus families and TE copies < 400 bases to produce a dataset satisfying the fixed quality criteria for RIPCAL. We also filtered out copies with less than 80% identity with the consensus sequence from the pairwise alignment and we excluded TE families with less than five sequences in the multiple alignments (TE consensus + four TE copies). In order to address the problem of degenerated and nested TE copies, the “long join procedure” implemented in the TEannot pipeline allow the connection of 2 ore more fragments to generate a TE copy.

### Analysis of GC content

The GC content of TE copies and sliding windows along the genome (GSW) were calculated an in-house script that divided the genome into sliding windows and calculated the GC percentage (GC%) in each window. R was used to calculate the distribution of TE copies and GSW according to the GC%.

### Search for proteins of the Dnmt1 family

We used RID, Masc1, Masc2 and Dim-2 protein sequences [14] as queries for Blastp [51] analysis to search for genes encoding cytosine DNA methytransferase from the Dnmt1 subfamily in the fungal genomes studied. We first searched for genes with GeneName = *RID* or GeneName = *Masc1* or GeneName = *Masc2* in Uniprot using SRS at EBI (http://srs.ebi.ac.uk/). Of the 14 genes found, we kept six non-redundant ones (O13369_ASCIM *Masc1 Ascobolus immersus*; O42731_ASCIM Masc2 *A. immersus*; B0B065_SORMA, *RID*, *Sordaria macrospora*; Q8NJV8_NEUTT, *RID*, *Neurospora tetrasperma*; Q8NJV9_NEUIN, *RID*, *Neurospora intermedia*; Q8NJW0_NEUCR, *RID*, *Neurospora crassa*). Using this set of six genes, we searched for similar genes in Uniprot with Blastp (e-value < 1e^−10^). We also searched the *Microbotryum* protein databank of the Broad Institute (http://www.broadinstitute.org/annotation/genome/Microbotryum_violaceum/MultiDownloads.html). We then filtered this new set of genes for redundancy particularly from re-sequenced species. We kept 30 non-redundant genes that were equally distributed along the tree. We then added the Dnmt1 genes found for the species in the tree (Additional file 1) to this set of genes, which gave a final list of 44 genes including all the *RID/Masc1* and *Masc2/Dim-2* genes of the 10 genomes of this study.

### Phylogenomic analysis of the 44 Dnmt1 fungal proteins

We performed an Interproscan analysis (Version 4.8 at http://www.ebi.ac.uk/Tools/pfa/iprscan/), [52] were to identify domains specific to each fungal Dnmt1 gene. The input parameters of this program were iprscan, Nocrc false, and GO terms true. The applications were blastprodom, fprintscan, hmmpir, hmmpfam, hmmsmart, hmmtigr, profilescan, hamap, patternscan, superfamily, signalp, tmhmm, hmmpanther, and gene3d (Additional file 5).

The PTHR10629 domain sequences from the Panther HMM domain database that were found in the studied genomes and other sequences from 15 ascomycetes and 8 basidiomycetes (including five and three of our study, respectively) were aligned with the T-Coffee multiple sequence alignment program [53] and analyzed phylogenetically with PhyML [54]. The analysis was performed on the Phylogeny.fr platform [55] and comprised the following steps: (i) sequences were aligned with T-Coffee (v6.85) [53] with the following pair-wise alignment methods: the 10 best local alignments (Lalign_pair) and an accurate global alignment (slow_pair). (ii) After alignment, ambiguous regions (i.e. those containing gaps and/or poorly aligned regions) were removed with Gblocks (v0.91b) [56] (using the following parameters: minimum length of a block after gap cleaning: 5, positions with a gap in less than 50% of the sequences were selected in the final alignment if they were within an appropriate block, all segments with contiguous, not conserved positions bigger than 8 were rejected, minimum number of sequences for a flank position: 55%. (iii) The phylogenetic tree was reconstructed using the maximum likelihood method implemented in the PhyML program (v3.0 aLRT) [57]. The WAG substitution model was selected assuming an estimated proportion of invariant sites (of 0.191) and four gamma-distributed rate categories to account for rate heterogeneity across sites. The gamma shape parameter was estimated directly from the data (gamma = 1.964). The reliability of the internal branch was assessed using the aLRT test (SH-Like). (iv) The phylogenetic tree was drawn and edited with TreeDyn software (v198.3) [58].

In addition we performed a MEME search to identify conserved motifs [59] (version 4.9.1) in the 44 proteins. The parameters used were -nmotifs 30, −minw 6 and -maxw 50. Of the 30 motifs found, we filtered out those present in at least one protein of the two subgroups of Dnmt1. Results (html files and motifs in logo format) are provided in Additional file 6.

## Additional files

Additional file 1:
**Is a figure representing a phylogenetic tree of species used in DNMT1 phylogenetic analysis.** Species used in RIP analysis are highlighted in blue. Genome name in Uniprot accession and abbreviation used in the manuscript (in blue) are in brackets. This tree was based on NCBI Taxonomy Browser (http://www.ncbi.nlm.nih.gov/Taxonomy/taxonomyhome.html). Only the topology is shown and the branch lengths are not proportional to evolutionary divergence time. Additional file 2:
**Is a figure representing a multiple sequence alignments (MSA) performed with T-Coffee (See Method section) for the C5_MTASE PFAM (PF000145) domain sequences extracted from the 44 Dnmt1 proteins used in the phylogenetic analysis.**
Additional file 3:
**Is a figure representing combined Block Diagrams found with MEME.** Eleven out of the 30 motifs found were filtered out because they were present at least once in both the Dnmt1 class I and class II subgroup (Figure 5A). Non-overlapping sites with a p-value below 0.0001. The height of the motif "block" is proportional to the log(p-value); height is truncated for motifs with a p-value of 1e-10. Additional file 4:
**Is a figure representing functional annotation of 44 fungal Dnmt1 proteins and phylogenetic analysis of the cytosine-specific methyltransferase domain.** This figure results from superposition of Figure 5 and Additional file 3. (A) Phylogenetic analysis of 44 cytosine-specific methyltransferase domains (PTHR10629): Gray rectangles in 5B, including PF00145, PS00094, PS00095 et PR00105) from Dnmt1 fungal proteins and *S. Pombe* DNMT2, which was used as an outgroup. (B) Functional annotation of genes (white rectangle) with Interproscan (Cf Methods section). PTHR10629:SF10 is drawn on top of PTHR10629 (the coordinates are the same). Where PTHR10629 is not visible, it was overlapped by PF00145. Combined Block Diagrams found with MEME are superposed on functional domains found with Interproscan. Additional file 5:
**Contains result of the interproscan analysis of proteins displayed in Figure** 5 **.**
Additional file 6:
**Contains html files corresponding to the results of the MEME analysis.** index.html is the main page to open. Display comments when mouse pass over a domain.
