# Supplementary figures and images for: Whole genome comparative analysis of transposable elements provides new insight into mechanisms of their inactivation in fungal genomes
Source: BMC Genomics. 2015 Feb 28;16(1):141. doi: 10.1186/s12864-015-1347-1 (PMC4352252; doi:10.1186/s12864-015-1347-1)

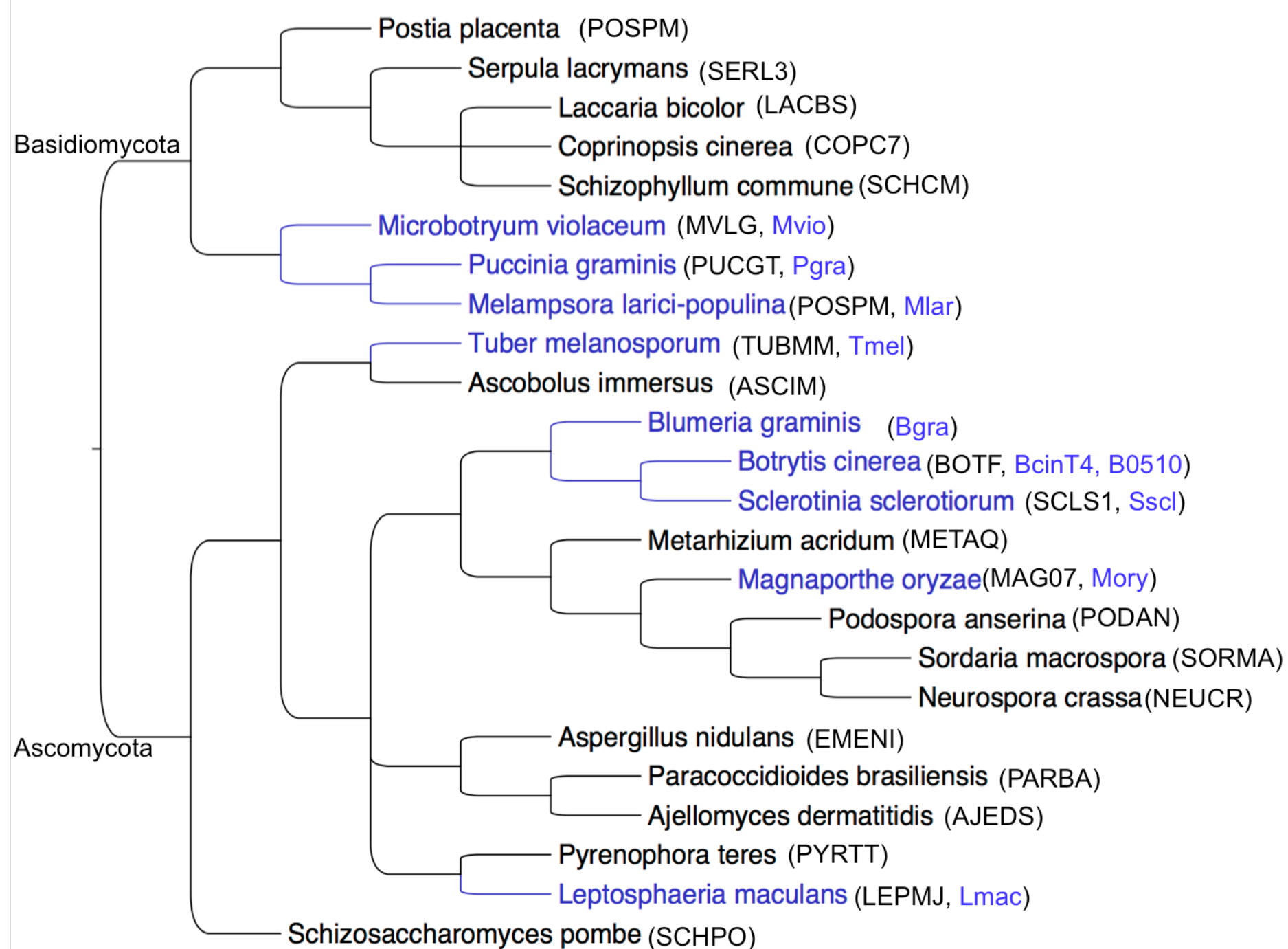

Supplement: Additional file 1: — Is a figure representing a phylogenetic tree of species used in DNMT1 phylogenetic analysis. Species used in RIP analysis are highlighted in blue. Genome name in Uniprot accession and abbreviation used in the manuscript (in blue) are in brackets. This tree was based on NCBI Taxonomy Browser (http://www.ncbi.nlm.nih.gov/Taxonomy/taxonomyhome.html). Only the topology is shown and the branch lengths are not proportional to evolutionary divergence time. [file 12864_2015_1347_MOESM1_ESM.pdf]

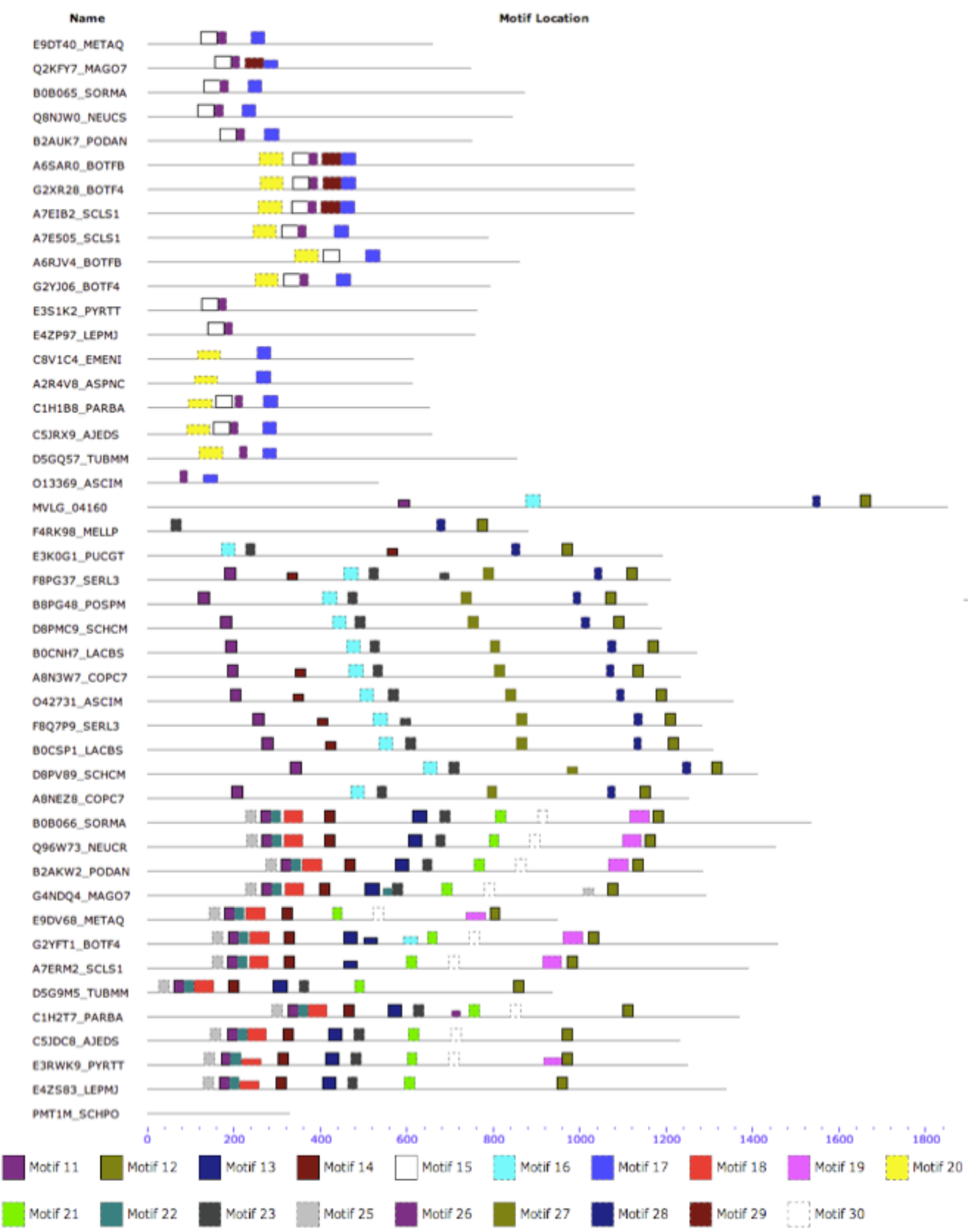

Supplement: Additional file 3: — Is a figure representing combined Block Diagrams found with MEME. Eleven out of the 30 motifs found were filtered out because they were present at least once in both the Dnmt1 class I and class II subgroup (Figure 5A). Non-overlapping sites with a p-value below 0.0001. The height of the motif "block" is proportional to the log(p-value); height is truncated for motifs with a p-value of 1e-10. [file 12864_2015_1347_MOESM3_ESM.pdf]

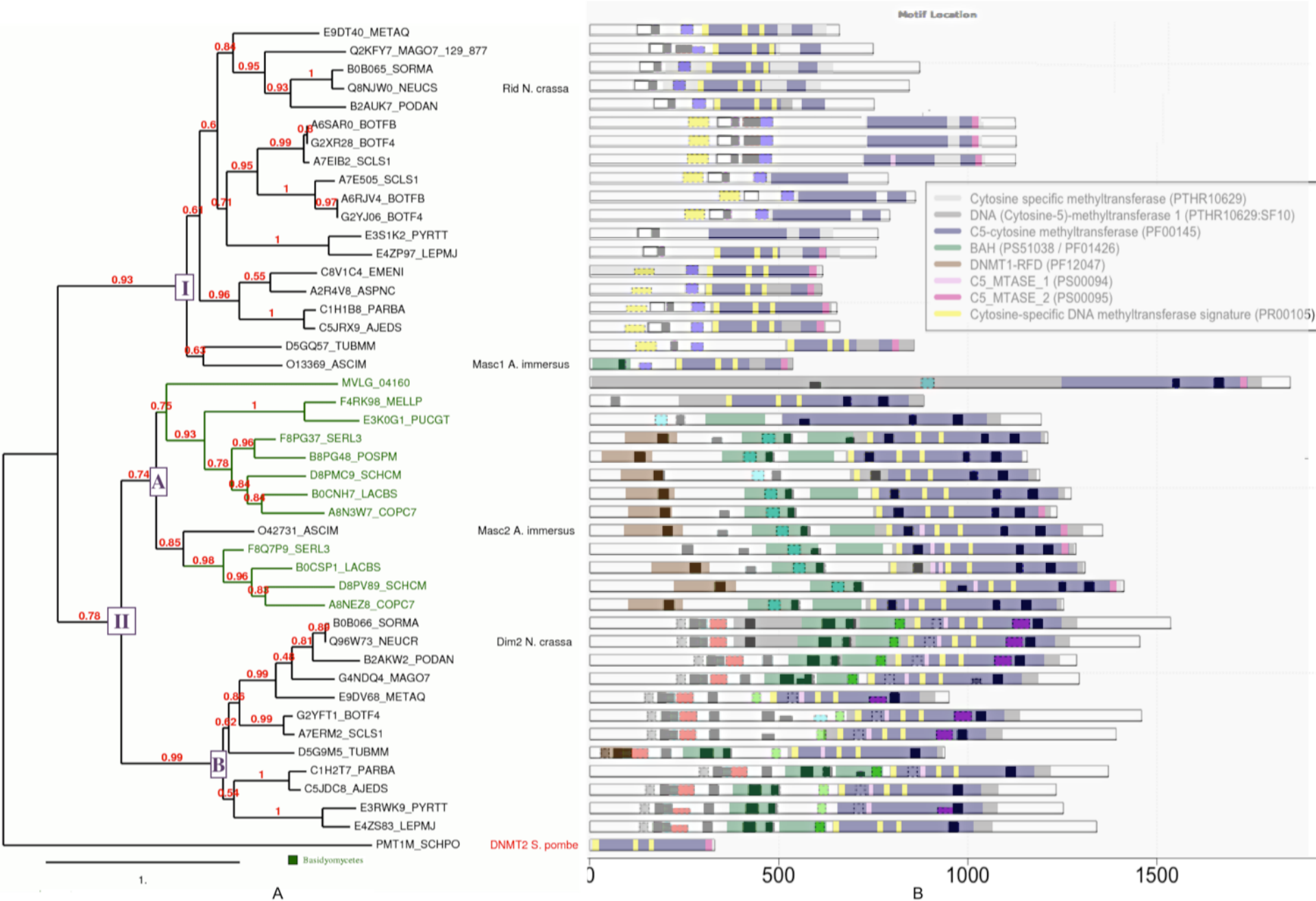

Supplement: Additional file 4: — Is a figure representing functional annotation of 44 fungal Dnmt1 proteins and phylogenetic analysis of the cytosine-specific methyltransferase domain. This figure results from superposition of Figure 5 and Additional file 3. (A) Phylogenetic analysis of 44 cytosine-specific methyltransferase domains (PTHR10629): Gray rectangles in 5B, including PF00145, PS00094, PS00095 et PR00105) from Dnmt1 fungal proteins and S. Pombe DNMT2, which was used as an outgroup. (B) Functional annotation of genes (white rectangle) with Interproscan (Cf Methods section). PTHR10629:SF10 is drawn on top of PTHR10629 (the coordinates are the same). Where PTHR10629 is not visible, it was overlapped by PF00145. Combined Block Diagrams found with MEME are superposed on functional domains found with Interproscan. [file 12864_2015_1347_MOESM4_ESM.pdf]

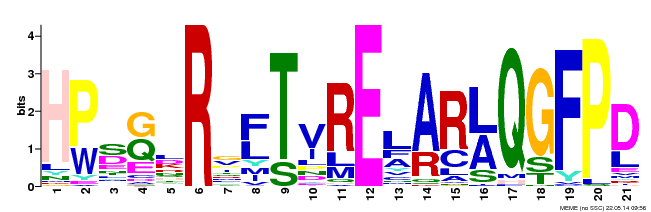

Supplement: Additional file 6: — Contains html files corresponding to the results of the MEME analysis. index.html is the main page to open. Display comments when mouse pass over a domain. [file 12864_2015_1347_MOESM6_ESM.zip › AdditionalFile6_MEME_Amselem__1941971504153245/logo1.png]

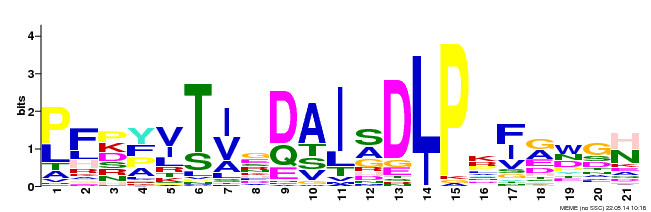

Supplement: Additional file 6: — Contains html files corresponding to the results of the MEME analysis. index.html is the main page to open. Display comments when mouse pass over a domain. [file 12864_2015_1347_MOESM6_ESM.zip › AdditionalFile6_MEME_Amselem__1941971504153245/logo10.png]

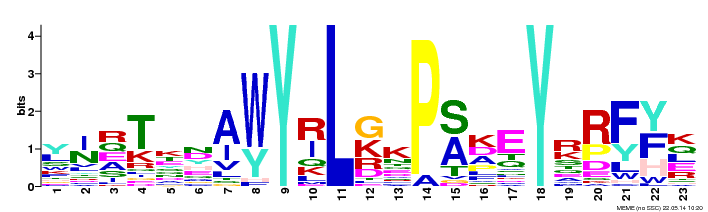

Supplement: Additional file 6: — Contains html files corresponding to the results of the MEME analysis. index.html is the main page to open. Display comments when mouse pass over a domain. [file 12864_2015_1347_MOESM6_ESM.zip › AdditionalFile6_MEME_Amselem__1941971504153245/logo11.png]

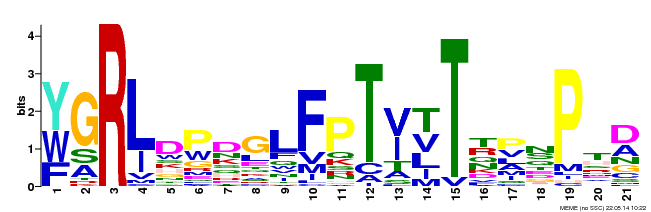

Supplement: Additional file 6: — Contains html files corresponding to the results of the MEME analysis. index.html is the main page to open. Display comments when mouse pass over a domain. [file 12864_2015_1347_MOESM6_ESM.zip › AdditionalFile6_MEME_Amselem__1941971504153245/logo12.png]

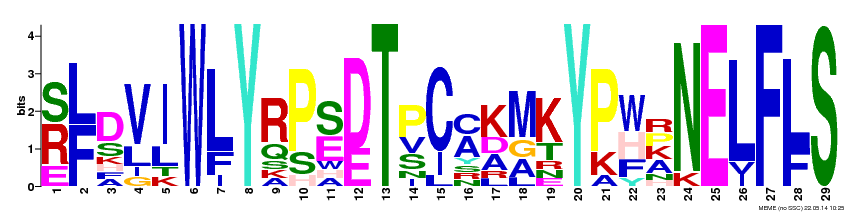

Supplement: Additional file 6: — Contains html files corresponding to the results of the MEME analysis. index.html is the main page to open. Display comments when mouse pass over a domain. [file 12864_2015_1347_MOESM6_ESM.zip › AdditionalFile6_MEME_Amselem__1941971504153245/logo13.png]

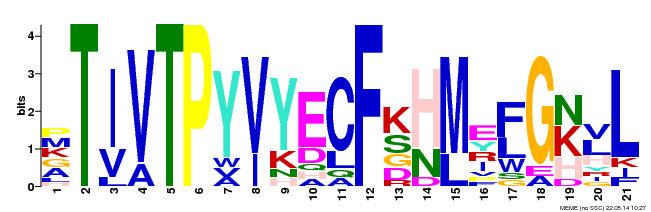

Supplement: Additional file 6: — Contains html files corresponding to the results of the MEME analysis. index.html is the main page to open. Display comments when mouse pass over a domain. [file 12864_2015_1347_MOESM6_ESM.zip › AdditionalFile6_MEME_Amselem__1941971504153245/logo14.png]

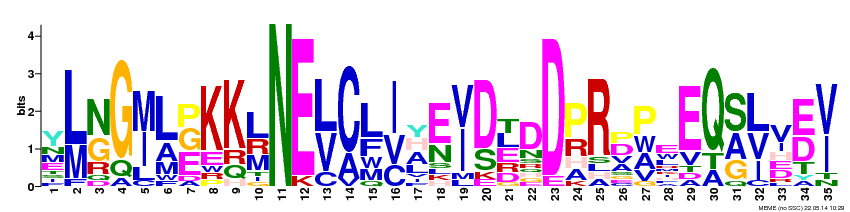

Supplement: Additional file 6: — Contains html files corresponding to the results of the MEME analysis. index.html is the main page to open. Display comments when mouse pass over a domain. [file 12864_2015_1347_MOESM6_ESM.zip › AdditionalFile6_MEME_Amselem__1941971504153245/logo15.png]

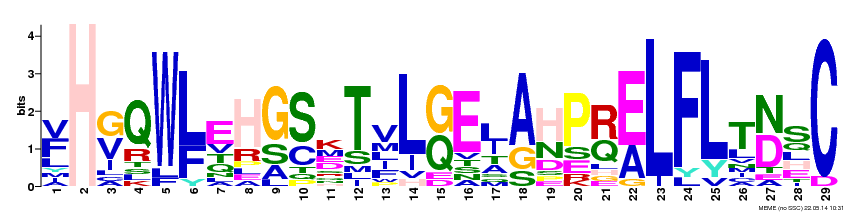

Supplement: Additional file 6: — Contains html files corresponding to the results of the MEME analysis. index.html is the main page to open. Display comments when mouse pass over a domain. [file 12864_2015_1347_MOESM6_ESM.zip › AdditionalFile6_MEME_Amselem__1941971504153245/logo16.png]

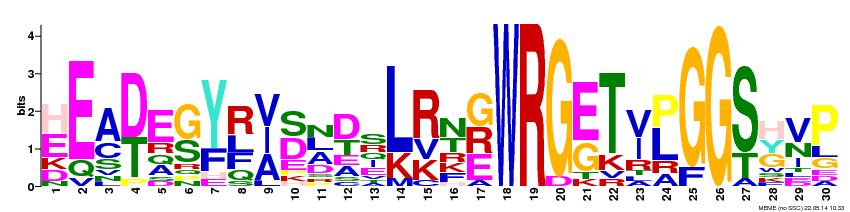

Supplement: Additional file 6: — Contains html files corresponding to the results of the MEME analysis. index.html is the main page to open. Display comments when mouse pass over a domain. [file 12864_2015_1347_MOESM6_ESM.zip › AdditionalFile6_MEME_Amselem__1941971504153245/logo17.png]

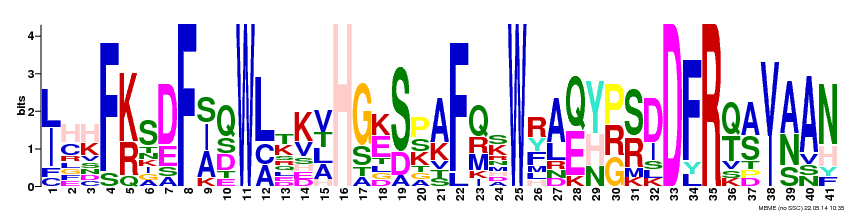

Supplement: Additional file 6: — Contains html files corresponding to the results of the MEME analysis. index.html is the main page to open. Display comments when mouse pass over a domain. [file 12864_2015_1347_MOESM6_ESM.zip › AdditionalFile6_MEME_Amselem__1941971504153245/logo18.png]

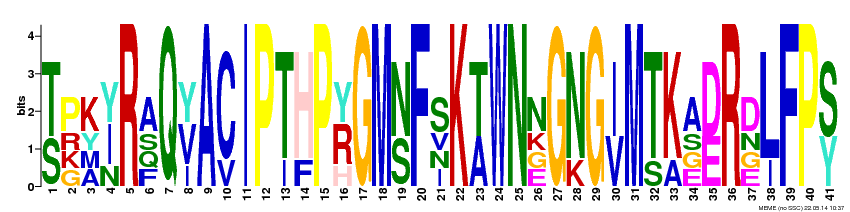

Supplement: Additional file 6: — Contains html files corresponding to the results of the MEME analysis. index.html is the main page to open. Display comments when mouse pass over a domain. [file 12864_2015_1347_MOESM6_ESM.zip › AdditionalFile6_MEME_Amselem__1941971504153245/logo19.png]

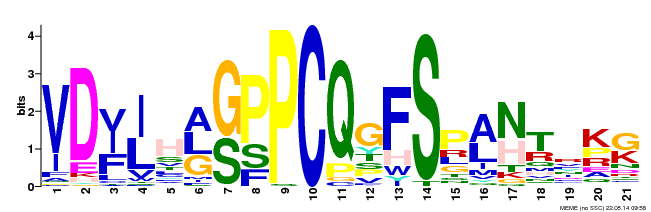

Supplement: Additional file 6: — Contains html files corresponding to the results of the MEME analysis. index.html is the main page to open. Display comments when mouse pass over a domain. [file 12864_2015_1347_MOESM6_ESM.zip › AdditionalFile6_MEME_Amselem__1941971504153245/logo2.png]

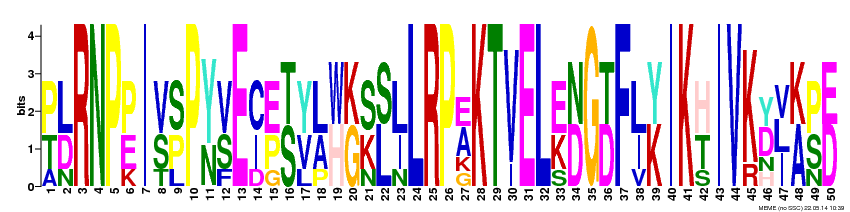

Supplement: Additional file 6: — Contains html files corresponding to the results of the MEME analysis. index.html is the main page to open. Display comments when mouse pass over a domain. [file 12864_2015_1347_MOESM6_ESM.zip › AdditionalFile6_MEME_Amselem__1941971504153245/logo20.png]

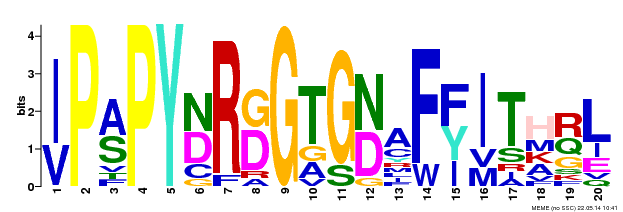

Supplement: Additional file 6: — Contains html files corresponding to the results of the MEME analysis. index.html is the main page to open. Display comments when mouse pass over a domain. [file 12864_2015_1347_MOESM6_ESM.zip › AdditionalFile6_MEME_Amselem__1941971504153245/logo21.png]

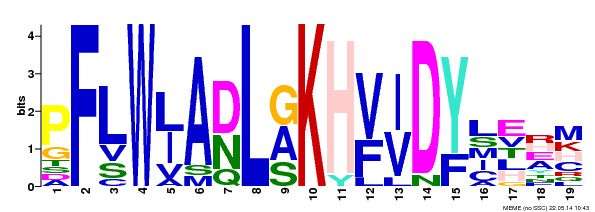

Supplement: Additional file 6: — Contains html files corresponding to the results of the MEME analysis. index.html is the main page to open. Display comments when mouse pass over a domain. [file 12864_2015_1347_MOESM6_ESM.zip › AdditionalFile6_MEME_Amselem__1941971504153245/logo22.png]

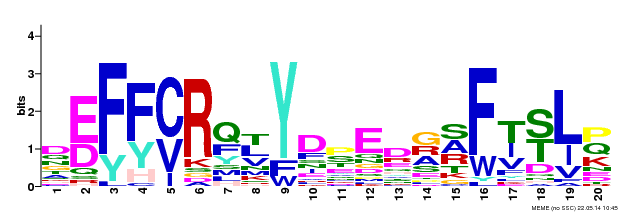

Supplement: Additional file 6: — Contains html files corresponding to the results of the MEME analysis. index.html is the main page to open. Display comments when mouse pass over a domain. [file 12864_2015_1347_MOESM6_ESM.zip › AdditionalFile6_MEME_Amselem__1941971504153245/logo23.png]

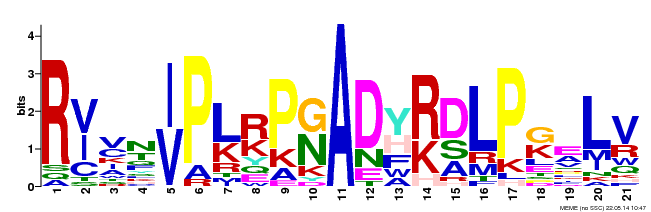

Supplement: Additional file 6: — Contains html files corresponding to the results of the MEME analysis. index.html is the main page to open. Display comments when mouse pass over a domain. [file 12864_2015_1347_MOESM6_ESM.zip › AdditionalFile6_MEME_Amselem__1941971504153245/logo24.png]

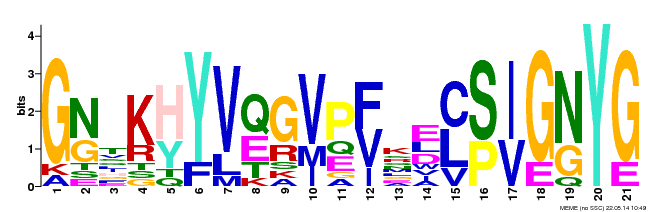

Supplement: Additional file 6: — Contains html files corresponding to the results of the MEME analysis. index.html is the main page to open. Display comments when mouse pass over a domain. [file 12864_2015_1347_MOESM6_ESM.zip › AdditionalFile6_MEME_Amselem__1941971504153245/logo25.png]

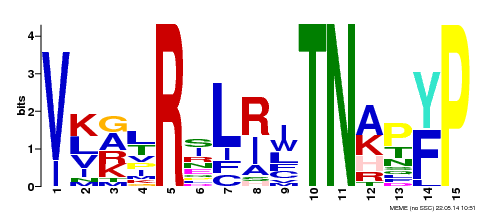

Supplement: Additional file 6: — Contains html files corresponding to the results of the MEME analysis. index.html is the main page to open. Display comments when mouse pass over a domain. [file 12864_2015_1347_MOESM6_ESM.zip › AdditionalFile6_MEME_Amselem__1941971504153245/logo26.png]

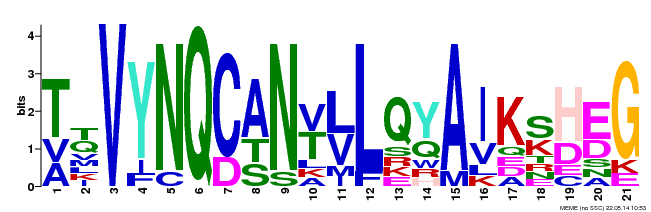

Supplement: Additional file 6: — Contains html files corresponding to the results of the MEME analysis. index.html is the main page to open. Display comments when mouse pass over a domain. [file 12864_2015_1347_MOESM6_ESM.zip › AdditionalFile6_MEME_Amselem__1941971504153245/logo27.png]

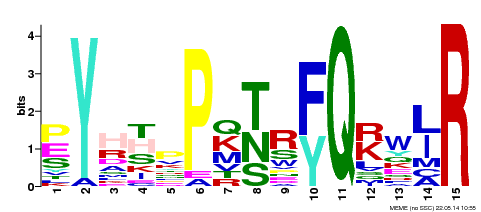

Supplement: Additional file 6: — Contains html files corresponding to the results of the MEME analysis. index.html is the main page to open. Display comments when mouse pass over a domain. [file 12864_2015_1347_MOESM6_ESM.zip › AdditionalFile6_MEME_Amselem__1941971504153245/logo28.png]

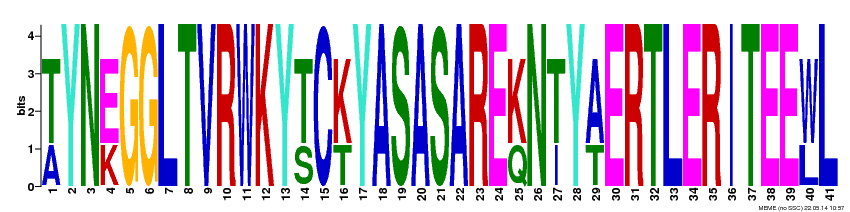

Supplement: Additional file 6: — Contains html files corresponding to the results of the MEME analysis. index.html is the main page to open. Display comments when mouse pass over a domain. [file 12864_2015_1347_MOESM6_ESM.zip › AdditionalFile6_MEME_Amselem__1941971504153245/logo29.png]

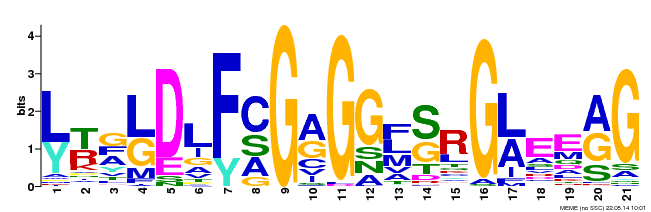

Supplement: Additional file 6: — Contains html files corresponding to the results of the MEME analysis. index.html is the main page to open. Display comments when mouse pass over a domain. [file 12864_2015_1347_MOESM6_ESM.zip › AdditionalFile6_MEME_Amselem__1941971504153245/logo3.png]

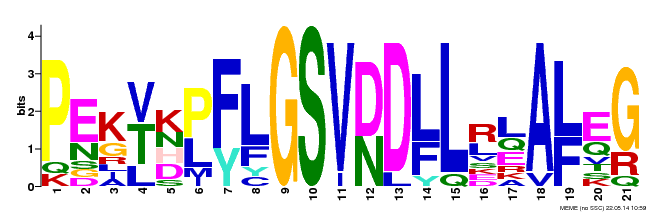

Supplement: Additional file 6: — Contains html files corresponding to the results of the MEME analysis. index.html is the main page to open. Display comments when mouse pass over a domain. [file 12864_2015_1347_MOESM6_ESM.zip › AdditionalFile6_MEME_Amselem__1941971504153245/logo30.png]

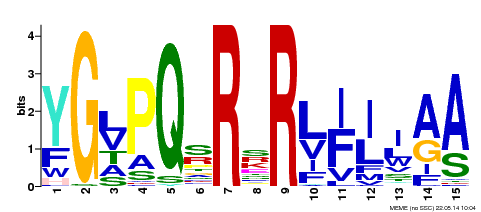

Supplement: Additional file 6: — Contains html files corresponding to the results of the MEME analysis. index.html is the main page to open. Display comments when mouse pass over a domain. [file 12864_2015_1347_MOESM6_ESM.zip › AdditionalFile6_MEME_Amselem__1941971504153245/logo4.png]

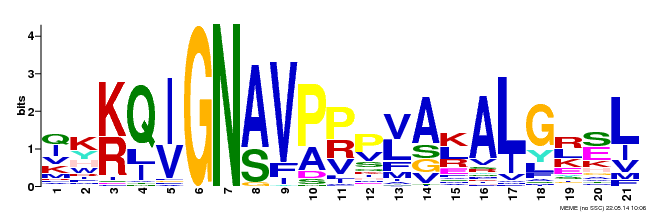

Supplement: Additional file 6: — Contains html files corresponding to the results of the MEME analysis. index.html is the main page to open. Display comments when mouse pass over a domain. [file 12864_2015_1347_MOESM6_ESM.zip › AdditionalFile6_MEME_Amselem__1941971504153245/logo5.png]

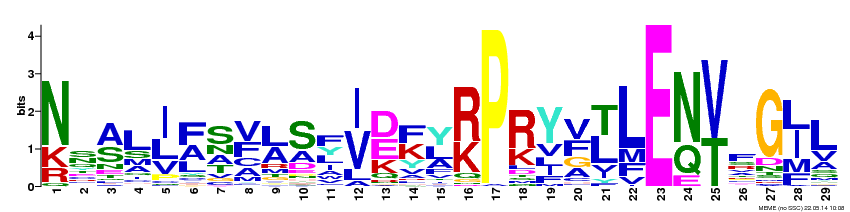

Supplement: Additional file 6: — Contains html files corresponding to the results of the MEME analysis. index.html is the main page to open. Display comments when mouse pass over a domain. [file 12864_2015_1347_MOESM6_ESM.zip › AdditionalFile6_MEME_Amselem__1941971504153245/logo6.png]

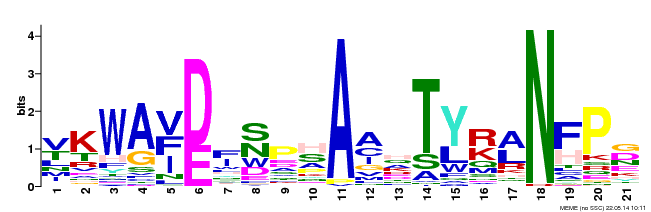

Supplement: Additional file 6: — Contains html files corresponding to the results of the MEME analysis. index.html is the main page to open. Display comments when mouse pass over a domain. [file 12864_2015_1347_MOESM6_ESM.zip › AdditionalFile6_MEME_Amselem__1941971504153245/logo7.png]

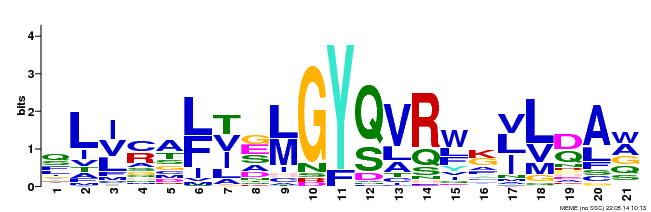

Supplement: Additional file 6: — Contains html files corresponding to the results of the MEME analysis. index.html is the main page to open. Display comments when mouse pass over a domain. [file 12864_2015_1347_MOESM6_ESM.zip › AdditionalFile6_MEME_Amselem__1941971504153245/logo8.png]

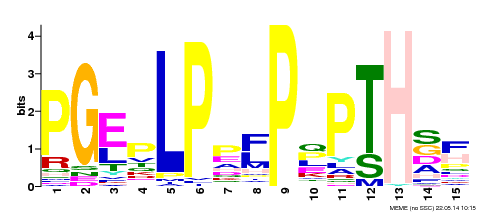

Supplement: Additional file 6: — Contains html files corresponding to the results of the MEME analysis. index.html is the main page to open. Display comments when mouse pass over a domain. [file 12864_2015_1347_MOESM6_ESM.zip › AdditionalFile6_MEME_Amselem__1941971504153245/logo9.png]

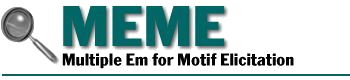

Supplement: Additional file 6: — Contains html files corresponding to the results of the MEME analysis. index.html is the main page to open. Display comments when mouse pass over a domain. [file 12864_2015_1347_MOESM6_ESM.zip › AdditionalFile6_MEME_Amselem__1941971504153245/meme.png]
