# Supplementary material for: Whole genome comparative analysis of transposable elements provides new insight into mechanisms of their inactivation in fungal genomes
Source: BMC Genomics. 2015 Feb 28;16(1):141. doi: 10.1186/s12864-015-1347-1 (PMC4352252; doi:10.1186/s12864-015-1347-1)
Supplement: Additional file 2: — Is a figure representing a multiple sequence alignments (MSA) performed with T-Coffee (See Method section) for the C5_MTASE PFAM (PF000145) domain sequences extracted from the 44 Dnmt1 proteins used in the phylogenetic analysis. [file 12864_2015_1347_MOESM2_ESM.pdf]

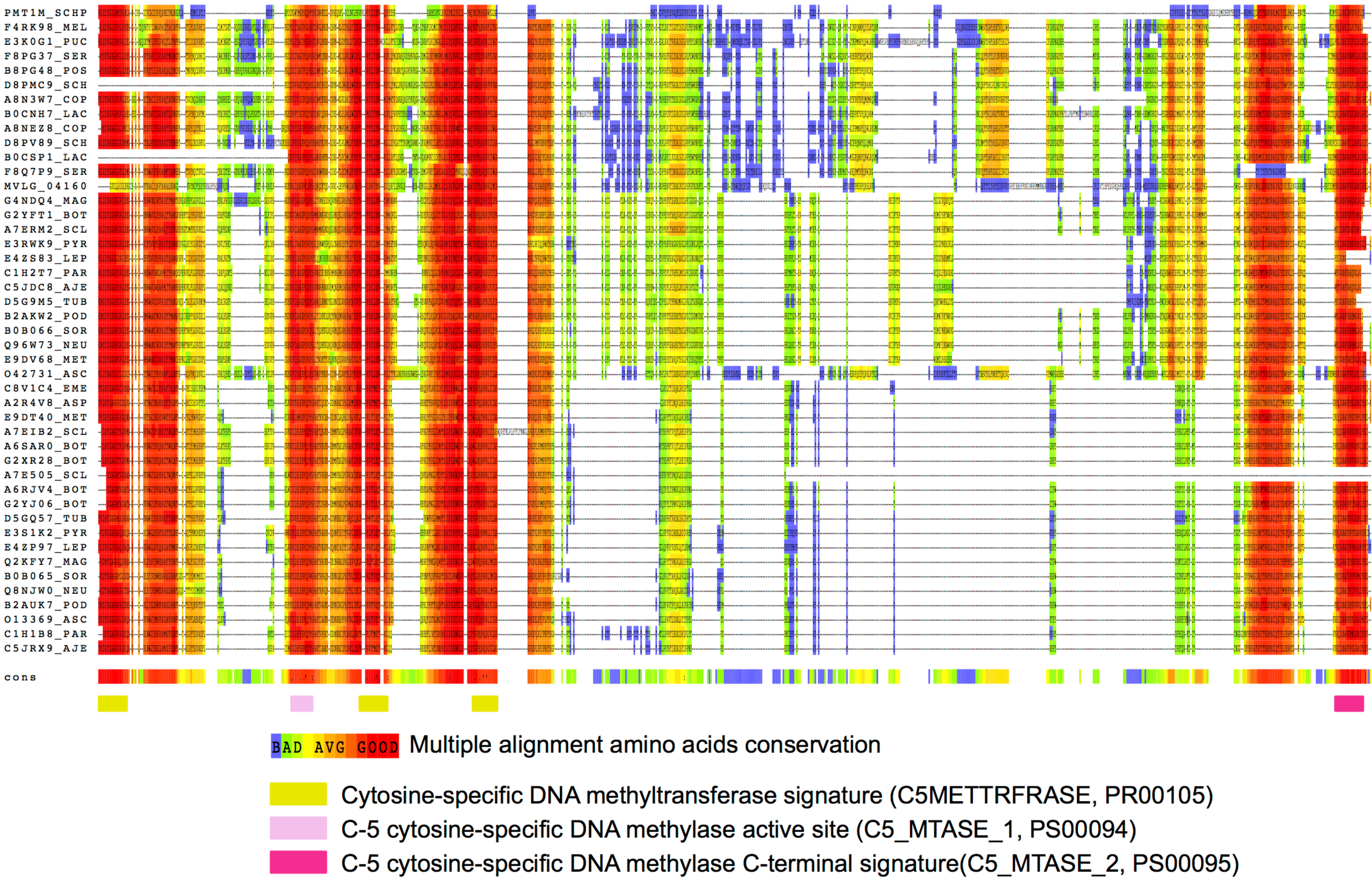
PMT1M_SCHP
F4RK98_MEL
E3K0G1_PUC
F8PG37_SER
B8PG48_POS
D8PMC9_SCH
A8N3W7_COP
B0CNH7_LAC
A8NEZ8_COP
D8PV89_SCH
B0CSP1_LAC
F8Q7P9_SER
MVLG_04160
G4NDQ4_MAG
G2YFT1_BOT
A7ERM2_SCL
E3RWK9_PYR
E4ZS83_LEP
C1H2T7_PAR
C5JDC8_AJE
D5G9M5_TUB
B2AKW2_POD
B0B066_SOR
Q96W73_NEU
E9DV68_MET
O42731_ASC
C8V1C4_EME
A2R4V8_ASP
E9DT40_MET
A7EIB2_SCL
A6SAR0_BOT
G2XR28_BOT
A7E505_SCL
A6RJV4_BOT
G2YJ06_BOT
D5GQ57_TUB
E3S1K2_PYR
E4ZP97_LEP
Q2KFY7_MAG
B0B065_SOR
Q8NJW0_NEU
B2AUK7_POD
O13369_ASC
C1H1B8_PAR
C5JRX9_AJE
cons
BAD AVG GOOD
Multiple alignment amino acids conservation
Cytosine-specific DNA methyltransferase signature (C5METTRFRASE, PR00105)
C-5 cytosine-specific DNA methylase active site (C5_MTASE_1, PS00094)
C-5 cytosine-specific DNA methylase C-terminal signature(C5_MTASE_2, PS00095)
