# Supplementary material for: Whole genome comparative analysis of transposable elements provides new insight into mechanisms of their inactivation in fungal genomes
Source: BMC Genomics. 2015 Feb 28;16(1):141. doi: 10.1186/s12864-015-1347-1 (PMC4352252; doi:10.1186/s12864-015-1347-1)
Supplement: Additional file 6: — Contains html files corresponding to the results of the MEME analysis. index.html is the main page to open. Display comments when mouse pass over a domain. [file 12864_2015_1347_MOESM6_ESM.zip › AdditionalFile6_MEME_Amselem__1941971504153245/DNMT1_MEME_19Motifs_Discriminants.html]

MEME

#### Combined Block Diagrams

Non-overlapping sites with a *p*-value better than 0.0001.  
The height of the motif "block" is proportional to -log(p-value), truncated at the height for a motif with a p-value of 1e-10.  
Click on any row to highlight sequence in all motifs. The motif blocks have tool tips with more information.

| Name | Combined *p*-value | Motif Location |
| --- | --- | --- |
 E9DT40\_METAQ | 9.48e-110 |  || Q2KFY7\_MAGO7 | 3.98e-119 |  |
| B0B065\_SORMA | 2.99e-135 |  |
| Q8NJW0\_NEUCS | 2.05e-135 |  |
| B2AUK7\_PODAN | 5.82e-121 |  |
| A6SAR0\_BOTFB | 6.24e-262 |  |
| G2XR28\_BOTF4 | 1.29e-262 |  |
| A7EIB2\_SCLS1 | 7.70e-240 |  |
| A7E505\_SCLS1 | 6.77e-168 |  |
| A6RJV4\_BOTFB | 2.57e-181 |  |
| G2YJ06\_BOTF4 | 5.28e-192 |  |
| E3S1K2\_PYRTT | 6.33e-96 |  |
| E4ZP97\_LEPMJ | 7.77e-90 |  |
| C8V1C4\_EMENI | 8.55e-124 |  |
| A2R4V8\_ASPNC | 5.92e-111 |  |
| C1H1B8\_PARBA | 7.50e-138 |  |
| C5JRX9\_AJEDS | 1.47e-135 |  |
| D5GQ57\_TUBMM | 2.87e-109 |  |
| O13369\_ASCIM | 2.73e-98 |  |
| MVLG\_04160 | 3.84e-89 |  |
| F4RK98\_MELLP | 2.25e-111 |  |
| E3K0G1\_PUCGT | 1.03e-118 |  |
| F8PG37\_SERL3 | 7.13e-186 |  |
| B8PG48\_POSPM | 6.09e-186 |  |
| D8PMC9\_SCHCM | 2.65e-151 |  |
| B0CNH7\_LACBS | 1.14e-176 |  |
| A8N3W7\_COPC7 | 1.44e-159 |  |
| O42731\_ASCIM | 4.14e-172 |  |
| F8Q7P9\_SERL3 | 1.09e-173 |  |
| B0CSP1\_LACBS | 2.40e-176 |  |
| D8PV89\_SCHCM | 1.94e-151 |  |
| A8NEZ8\_COPC7 | 1.23e-171 |  |
| B0B066\_SORMA | 0.00e+00 |  |
| Q96W73\_NEUCR | 0.00e+00 |  |
| B2AKW2\_PODAN | 8.89e-260 |  |
| G4NDQ4\_MAGO7 | 1.35e-234 |  |
| E9DV68\_METAQ | 1.58e-196 |  |
| G2YFT1\_BOTF4 | 0.00e+00 |  |
| A7ERM2\_SCLS1 | 1.68e-263 |  |
| D5G9M5\_TUBMM | 5.30e-219 |  |
| C1H2T7\_PARBA | 4.15e-235 |  |
| C5JDC8\_AJEDS | 5.96e-240 |  |
| E3RWK9\_PYRTT | 6.04e-172 |  |
| E4ZS83\_LEPMJ | 1.39e-177 |  |
| PMT1M\_SCHPO | 3.00e-26 |  |
||  |  |  |
| --- | --- | --- |
|  | | 0          200          400          600          800          1000          1200          1400          1600          1800 |

|  |  |
| --- | --- |
|  | Motif 11 |

|  |  |
| --- | --- |
|  | Motif 12 |

|  |  |
| --- | --- |
|  | Motif 13 |

|  |  |
| --- | --- |
|  | Motif 14 |

|  |  |
| --- | --- |
|  | Motif 15 |

|  |  |
| --- | --- |
|  | Motif 16 |

|  |  |
| --- | --- |
|  | Motif 17 |

|  |  |
| --- | --- |
|  | Motif 18 |

|  |  |
| --- | --- |
|  | Motif 19 |

|  |  |
| --- | --- |
|  | Motif 20 |

|  |  |
| --- | --- |
|  | Motif 21 |

|  |  |
| --- | --- |
|  | Motif 22 |

|  |  |
| --- | --- |
|  | Motif 23 |

|  |  |
| --- | --- |
|  | Motif 25 |

|  |  |
| --- | --- |
|  | Motif 26 |

|  |  |
| --- | --- |
|  | Motif 27 |

|  |  |
| --- | --- |
|  | Motif 28 |

|  |  |
| --- | --- |
|  | Motif 29 |

|  |  |
| --- | --- |
|  | Motif 30 |
